# Modulating the Activity of the VMPFC With tDCS Alters the Social Framing Effect

**DOI:** 10.3389/fnbeh.2021.677006

**Published:** 2021-08-26

**Authors:** Yuyou Chen, Xinbo Lu, Ping Yu, Lulu Zeng, Hang Ye, Qing Shi, Wenmin Guo

**Affiliations:** ^1^Center for Economic Behavior and Decision-Making, Zhejiang University of Finance and Economics, Hangzhou, China; ^2^School of Economics, Zhejiang University of Finance and Economics, Hangzhou, China; ^3^School of Economics, Jiaxing University, Jiaxing, China; ^4^School of Information Management and Artificial Intelligence, Zhejiang University of Finance and Economics, Hangzhou, China

**Keywords:** social framing effect, transcranial direct current stimulation, harm frame, help frame, ventromedial prefrontal cortex

## Abstract

Numerous experimental studies have replicated the social framing effect-the observation that people’s decisions related to economic benefits and feelings depend on the method of presentation. Previous neuroimaging studies have shown that the ventromedial prefrontal cortex (VMPFC) plays a part in the influence of framing and how individuals think about the feelings of others. Based on this, we used transcranial direct current stimulation (tDCS) to modulate neuronal activity in the VMPFC to determine the likelihood of a direct association between VMPFC activity and the social framing effect. Subsequently, in three stimulation treatments, we assessed the presence of the social framing effect, as demonstrated by a disparity between harm degree and help degree. The findings revealed a social framing effect in the participants in the control group and the sham treatment but no social framing effect in the participants in the anodal or cathodal treatments. Furthermore, sex differences were observed in the sham treatment’s social framing effect, whereas no sex differences were observed in the anodal or cathodal treatments. The participants tended to harm the victim after receiving anodal or cathodal tDCS over the VMPFC and did not change their helping behaviour in any stimulations. Consequently, a clear causal link between the behaviour of the VMPFC and the social framing effect was found in the present research.

## Introduction

Human decisions are always influenced by the way information is presented. Equivalent information can be presented with positive or negative connotations depending on what features are highlighted. This phenomenon is called the framing effect. The framing effect deviates from standard economic assumptions (Tversky and Kahneman, 1981) and has become a popular research topic, particularly cognitive psychology, linguistics and discourse analysis (Tannen, 1993), communication and media studies (Pan and Kosicki, 1993; Scheufele, 1999), and political science and policy studies (Shön and Rein, 1994; Triandafyllidou and Fotiou, 1998; Benford and Snow, 2000). Individuals tend to avoid risks when a positive frame is presented but seek risks when a negative frame is presented (Tversky and Kahneman, 1981). This phenomenon is a within-subjects risky-choice framing problem (Mahoney et al., 2011), which is produced by within-subjects rather than inter-subjects risk choice. Prospect theory shows that a loss is more significant than the equivalent gain (Tversky and Kahneman, 1981), that a certain gain is favoured over a probabilistic gain and that a probabilistic loss is preferred to a determined loss (Tversky and Kahneman, 1981). Framing research is not homogeneous; instead, researchers have proposed various taxonomies. For example, Peng et al. (2013) researched five types of framing effects in a medical situation, including the goal framing effect, the attribute framing effect, the risky choice framing effect with options equivalent, the risky choice framing effect with options not equivalent and the number size framing effect. The social framing effect may be first proposed by Ellingsen et al. (2012), found that behaviour is more likely to be cooperative when the prisoners’ dilemma game is called the community game than when it is called the stock market game. Recently, Liu et al. (2020) distinguished the social framing effect from the nonsocial effect according to whether a social dilemma between oneself and others is involved in the scenario. In their opinion, a social framing effect manifests when changing the description of a social dilemma significantly modulates the preference of decision-makers for various options. In contrast, nonsocial framing, such as gamble framing, is thought to occur, so people can maximize the utility of their choices to be more beneficial or less risky.

However, the research on the effect of the social framing effect is insufficient thus far, and fewer researchers have investigated the neural mechanism of this behaviour. Liu et al. (2020) found that the social framing effect was associated with stronger activation in the temporoparietal junction (TPJ), especially the right part (r-TPJ), and became more prominent under anodal transcranial direct current stimulation (tDCS). In the resting state, there is a significant positive correlation between the amplitude of the low-frequency fluctuation (ALFF) value of the right temporoparietal junction (r-TPJ) and the strength of the functional connectivity value between the medial prefrontal lobe and the caudate within a moral network, which can effectively predict the social framing effect (Fang et al., 2021). These studies are all about the influence of the rTPJ on the social framing effect, which can strengthen the social framing effect. It is natural to think about whether brain regions are also involved in the social framing effect and attenuate this effect.

Over approximately the last decade, there has been a surge of research on the framing effect’s neural basis. The right orbitofrontal cortex (r-OFC) and the ventromedial prefrontal cortex (VMPFC) are significantly correlated with decreased susceptibility to the framing effect and enhanced activity (De Martino et al., 2006). Deppe et al. (2005) found that individual activity changes in the VMPFC during judgements are correlated with the degree of an individual’s susceptibility to framing information. For the framing effects associated with gain and loss options, the brain’s prefrontal and parietal cortices are involved in working memory and imagery in the selection process (Gonzalez et al., 2005). Activation in the amygdala is modulated by the framing effect (Talmi et al., 2010). In small group contexts, the insula and parietal lobe in the right hemisphere are distinctively activated, while framing effects are diminished (Zheng et al., 2010). Silveira et al. (2015) found that mental frames, particularly social value labels, modulate sensory processing’s cognitive and affective aspects and involve the bilateral anterior cingulate cortex and temporoparietal junction. When a close friend provides feedback, an individual’s susceptibility to the framing effect is modulated by the valence of social feedback, which activates the VMPFC and posterior cingulate cortex (Sip et al., 2015). VMPFC activity has been observed for non-costly social decisions (Kuss et al., 2015). In short, the framing effect involves multiple brain regions, among which the VMPFC appears to be commonly involved, and there is a correlation between the framing effect and the VMPFC.

Previous studies have shown that the VMPFC is correlated with the framing effect and impacts a variety of behaviours. The social framing effect may involve individual social decision-making, including moral decision-making, empathy, fear, etc. In both rodents and humans, the VMPFC is implicated in social processing, cortical regulation of anxiety, and safety learning (Milad and Quirk, 2002; Adolphs, 2010; Hartley and Phelps, 2010; Fossati, 2012; Meyer-Lindenberg and Tost, 2012; Van Kerkhof et al., 2013; Sangha et al., 2014; Van Kerkhof et al., 2014). Lesions of the VMPFC have been shown to blunt normal emotional responses (Hernandez et al., 2009). Activation of the VMPFC is associated with learned suppression of fear and anxiety during cognitive-behavioural training and extinction of conditioned fear (Milad and Quirk, 2002; Phelps et al., 2004; Schiller et al., 2008; Hartley and Phelps, 2010; Sotres-Bayon and Quirk, 2010; Eisenberger et al., 2011; Kim et al., 2011). Patients with VMPFC damage experience impaired empathy, poor decision-making, and “moral character” deterioration because they are unable to generate the emotions that guide adaptive decision-making in healthy individuals (Bechara et al., 1996; Anderson et al., 1999; Shamay-Tsoory, 2007). The VMPFC might be involved in the extinction of arousal caused by emotional stimuli (Nejati et al., 2021). Activity in the VMPFC is correlated with goal values independent of self-control (Hare et al., 2009). Patients with VMPFC damage report significantly less confidence in the dictatorial game and the trust game, indicating that VMPFC is essential in altruistic and trust decisions (Krajbich et al., 2009; Moretto et al., 2013). Activations of the VMPFC are related to affective empathy (Hynes et al., 2006; Saxe, 2006; Vollm et al., 2006; Mobbs et al., 2009), particularly to empathy for positive emotions (Morelli et al., 2014). Evidence implicates the VMPFC and broader medial frontal cortex in the transition from acute to chronic pain, specifically *via* altered functional connectivity with emotion and reward circuitry (Baliki et al., 2012; Hashmi et al., 2013). The VMPFC is an integrative hub for emotional, sensory, social, memory, and self-related information processing (Roy et al., 2012). The VMPFC region appears to be involved in evaluating the long–term benefits of cooperative relationships, valuing abstract incentives such as helping anonymous others through charitable donations and regulating emotional reactions that could jeopardize valued relationships (Rilling and Sanfey, 2011). Emotional and utilitarian appraisals are computed independently and in parallel and passed to the VMPFC, where they are integrated into an overall moral value judgement (Hutcherson et al., 2015). Difficult moral decisions activate the bilateral TPJ and deactivate the VMPFC and OFC (Feldmanhall et al., 2014). Extending from these studies, the VMPFC may be one of the brain regions that affect the social framing effect.

Another noteworthy issue is that there are gender differences in social framing. In a one-shot prisoners dilemma experiment, female participants were more sensitive to the social frame than male participants when the dilemma was called the community game than when it was called the stock market game (Ellingsen et al., 2013). Therefore, in this study, we will pay more attention to gender differences in social framing effects.

Neuroimaging studies are useful in establishing correlations; however, these studies did not demonstrate a direct causal relation between the brain region and behaviour. Noninvasive brain stimulation techniques, such as tDCS, can be useful for addressing this question and make it possible to detect its effect on behaviour more accurately. tDCS has been demonstrated to modulate cognitive functions by changing cortical excitability (Kuo et al., 2014; Lefaucheur et al., 2017). Generally, anodal tDCS enhances cortical excitability, whereas cathodal stimulation restrains it (Nitsche and Paulus, 2000).

In the current study, using tDCS, we aimed to investigate whether modulating VMPFC excitability can directly influence the social framing decisions of our participants. We recruited 192 people to participate in the experiment, of which 189 completed the social framing experiment. We hypothesized that stimulation in the VMPFC would change the decisions of the participants in the social framing experiment. Our experiment consisted of two tasks-a help frame task and a harm frame task-in which participants made a trade-off between the economic benefits and the feelings of others across the three treatments. If the participants preferred income maximisation in the harm frame task, their partners would increasingly receive a painful shock. In addition, in the help frame task, the participants were asked to decide whether to help their partners at a cost to themselves or to pay nothing and let their partners receive a shock. We adopted a within-subjects design to test the social framing effect. In addition to the three stimulation types (anodal, cathodal, and sham) used to test the stimulation effect, a control group without any manipulation was conducted to support the claims on the sham group. In general, this research aimed to investigate the causal relationship between the VMPFC and the effect of social framing to explore the neural mechanism of the social framing effect.

## Materials and Methods

### Subjects

A total of 192 healthy college students were recruited randomly to participate in our experiment. Three participants were excluded due to being left-handed, leaving 189 participants (control group: 24 females and 22 males, mean age of 20.67 years, varying from 18 to 25 years old; stimulation groups: 73 females and 70 males, mean age of 20.03 years, varying from 17 to 30 years old) in the final sample. The following criteria were met by 189 participants: unfamiliar with tDCS; right-handed, and no records of psychiatric disability, psychological diagnosis, or cognitive impairment. We conducted* a priori* sample size calculations *via* G·Power 3.1 (Faul et al., 2009). The results showed that based on a medium effect size (*f* = 0.40), an alpha level of 0.05, power of 0.95 and three groups, the required sample size was 100. Before the experiment, each participant was fully informed of the possible side effects of tDCS, and they were asked to report any discomfort after the experiment. The entire experiment lasted approximately 40 min, and each participant received a pay-out of approximately 26.47 CNY (approximately 4.08 US dollars) for completing the tasks, with compensation ranging from 25 CNY to 30 CNY, depending on their performance and the computer program. Furthermore, the participants received a presentation fee of 10 CNY (approximately 1.55 US dollars) during the social framing task. In this experiment, no participants reported physical discomfort. Demographic information is summarized in Table 1.

**Table 1:** Demographic characteristics of participants.

Variables	Group	Control	Sham	Anodal	Cathodal
Sample size (*n*)		46	47	48	48
Age	Mean	20.67	20.28	19.92	19.92
Gender	Female (male)	24 (22)	26 (21)	24 (24)	23 (25)
AFI	Mean	2.28	2.00	1.96	1.88
MC	Mean	2.22	2.30	2.19	2.10

*Note: AFI = annual family income; MC = monthly consumption; AFI denotes categorical variable (1 to 6): (1) 0 to ¥100,000, (2) ¥100,000 to ¥200,000, (3) ¥200,000 to ¥300,000, (4) ¥300,000 to ¥400,000, (5) ¥400,000 to ¥500,000, (6) ¥500,000 or over; MC denotes categorical variable (1–6) : (1) 0 to ¥1,000, (2) ¥1,000 to ¥2,000, (3) ¥2,000 to ¥3,000, (4) ¥3,000 to ¥4,000, (5) ¥4,000 to ¥5,000, (6) ¥5,000 or above*.

Before entering the study, participants provided verbal, informed consent, and the thesis was accepted by the Zhejiang University of Finance and Economics Ethics Committee. No participants indicated any scalp irritation or headaches as a negative side effect. The ethics number of this experiment was 20210104.

### tDCS

For tDCS, a mild direct current was applied to the scalp by means of two saline-soaked surface sponge electrodes. A Bluetooth system-controlled battery-driven stimulator supported a steady current (multichannel, noninvasive wireless tDCS neurostimulator, Starlab, Barcelona, Spain). It was tweaked to increase cortical excitability in the target region while causing no physiological harm to the subjects. In general, cortical excitability is increased when anodal stimulation is used, but it is reduced when cathodal stimulation is used (Nitsche and Paulus, 2000).

**Figure 1:**
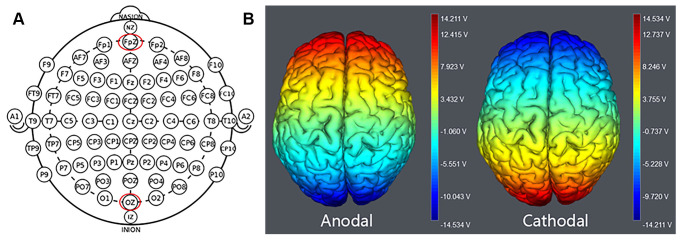
**(A)** Electrodeposition schematics and positions. **(B)** Activation modes of two successful treatments with tDCS. The axis of this potential electronic diagram represents the range of input voltages. The red colour indicates the strongest electrical field over Fpz and Oz.

Each of the three stimulation treatments was randomly allocated to the participants: anodal tDCS (*n* = 48, 24 females and 24 males) over the VMPFC, cathodal tDCS (*n* = 48, 23 females and 25 males) over the VMPFC, and sham tDCS (*n* = 47, 26 females and 21 males). In accordance with the international EEG 10-20 system, the anodal electrode (3 cm * 3 cm) was positioned over the Fpz for anodal stimulation, while the cathodal electrode (5 cm * 7 cm) was placed over the Oz. The anodal electrode (5 cm * 7 cm) was placed over Oz, while the cathodal electrode (3 cm * 3 cm) was placed over Fpz to achieve cathodal stimulation (Figures 1A,B). For sham stimulation, the procedures were the same, but the current lasted only for the first 30 s. Although the subjects could have sensed the scratching at first, there was no current present for the remainder of the stimulus. This method has proven reliable because cortical excitability is not modulated by such limited stimulation; however, participants do experience an initial itch and conclude that they had received stimulation (Gandiga et al., 2006). The current was steady, the amplitude was 1.5 mA (Dymond et al., 1975) with a ramp up and down of 30 s, and its protection and reliability were shown in previous experiments (Nitsche and Paulus, 2000; Nitsche et al., 2003). During the experiment, the device parameters were set according to the type of stimulus received, and the other processes were completely the same, which ensured that the participants did not know the type of stimulus they received during the experiment. The electrode montage and tDCS parameters were identical to those that successfully modulated cortical excitability of the VMPFC in previous studies (Zheng et al., 2016; Li et al., 2020).

### Experimental Task and Procedure

#### Experiment

The social framing experiment included two tasks: a help frame task and a harm frame task. Each task had two phases: an instruction phase and a decision-making phase. In the instruction phase, the subjects were familiarised with the decision-making phase and given several practice trials. They were also told that they would not receive feedback concerning their decisions during the task but would instead receive a sum proportional to their total winnings at the end of the experiment. The experiment design was the same as that used in the previous study (Liu et al., 2020).

Before making a decision, each participant was told that their role in the experimental task would be determined by a lottery and that one role is the “decision-maker” and the other is the “victim.” It further stated that the victim will conduct the experiment in another room. However, we secretly manipulated the lottery so that the real participants always play the role of “decision-maker.” Therefore, in reality, no participant served as a “victim.” In the decision-making phase of the two tasks, each participant was shown a horizontal slider with photos and texts on its left and right ends. One consequence was the “Help frame”, which read “help the other person to avoid experiencing a severe shock and deduct 5 CNY from your own payment.” condition. In contrast, the alternate outcome was “do not prevent the other person from being shocked and keep your entire payment.” The “Harm frame,” which stated “harm the other person by administering a painful shock and keep all your payment” or “do not harm the other person by administering a shock and deduct 5 CNY from your own reward,” was the alternative consequence. Each participant faced virtually the same “costly helping” decision in both frame situations. Only the way the outcomes were stated differently between the two scenarios. In the “Help frame” condition, the outcome “the participant keeps all money, and the victim suffers a severe shock” was represented as not helping others, but in the “Harm frame” condition, it was portrayed as purposefully injuring others. In the “Help frame” condition, the outcome “the participant loses 5 CNY and the victim suffers no shock” was portrayed as helping others, whereas in the “Harm frame” condition, it was described as avoiding injuring others. To show his/her relative choice between the two possible outcomes, each participant was asked to move the slider. The likelihood of the two potential outcomes was decided by the slider’s ultimate positioning. The participants were told during the task instruction phase that they could move the slider to change the odds of the two outcomes; if they did not want to change the probabilities, they may keep the slider at 50%. This phase of the social frame consisted of two runs of two trials lasting ~5 min.

#### Experimental Procedure

**Figure 2:**
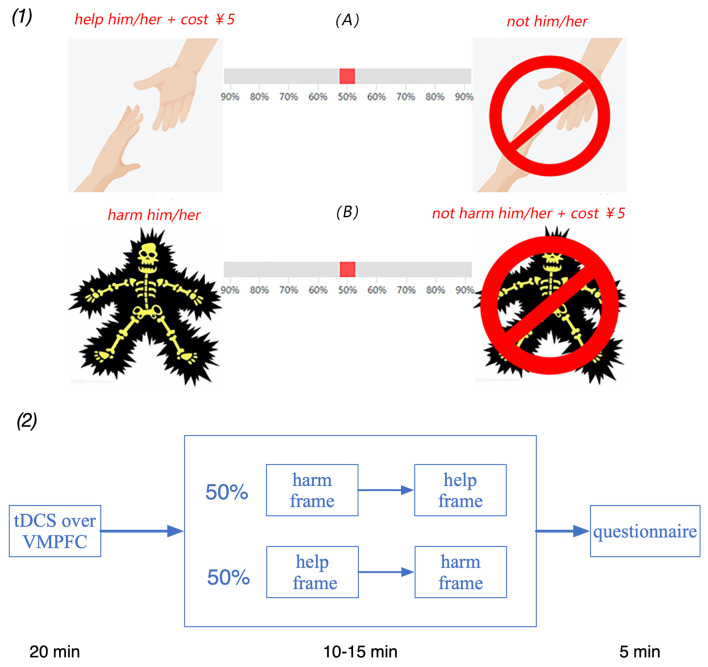
(1) Experimental design of the social frame task. (2) Schematic representation of the configuration of experiments. The participant was asked to complete the social frame task, including the harm frame and the help frame, after 20 min of stimulation.

The software o-Tree (Chen et al., 2016) was used to introduce the tasks and calculate the final payoff automatically in the experiment. The whole experiment was carried out in three steps (Figure 2). First, each participant was exposed to one of the three stimulation modes for 20 min. Second, the participants were asked a control question to test whether they fully understood the profitable implications of their decision. The participants were informed of how their decisions influenced their final payments. Then, the participants were asked to decide which choice they wanted to increase their money in the two frame tasks. Finally, the participants completed a questionnaire before the final payment was received. Personal information issues, such as age, sex, salary, and consumer spending, were included in the questionnaire. The participants were informed of how their decisions influenced their final payments.

To avoid the order effect, each participant was randomly divided into group A (*n* = 72, 36 females and 36 males) and group B (*n* = 71, 38 females and 33 males). Participants in group A completed the harm frame task and the help frame task successively, and participants in group B completed the help frame task and the harm frame task successively. The harm frame task and the help frame task were presented only one time in the decision-making phase.

### Data Analysis

Under the social frame, the subjects faced dilemmas of the same nature but with different descriptions. The framing effect was measured by the difference between the subject’s tendency to sacrifice his/her interests to avoid the pain of another (i.e., the tendency to help others) under the two frames. Specifically, under the harm frame, when the participant chose the option closest to the end of “Do not harm him/her + cost 5 CNY,” 9 points were awarded; fewer points were awarded as the choice moved towards the other end, with the option closest to the “harm him/her” end awarded only 1 point. Correspondingly, under the help frame, when the participant chose the option closest to the “help him/her avoid injury + cost 5 CNY”, 9 points were awarded. The following was used to calculate the frame effect score.

Frame Effect Score = [Help Degree in Harm Frame − Help Degree in Help Frame]/[Help Degree in Harm Frame + Help Degree in Help Frame]

First, we concentrated on a test of whether there was a social framing effect across three stimulation conditions. Because the frame was designed as a within-subjects factor, the harm degree and the help degree were no independent samples, the Wilcoxon test was applied to analyse the difference between the harm degree and the help degree across three stimulations.

Second, we conducted an ANOVA with tDCS stimulation type (anodal, cathodal, and sham) as a “between-subjects” factor and the frame effect scores as dependent variables to test the stimulation effect. If a significant main effect appeared in the social framing effect among the three stimulation conditions (anodal, cathodal, and sham), *post hoc* analyses (Bonferroni) within ANOVA were run to clarify the stimulation effect.

Last, to explain the difference in the social frame effect under the three stimulation conditions, we took an ANOVA with tDCS stimulation type (anodal, cathodal, and sham) as a “between-subjects” factor and the harm degrees (the help degrees) as dependent variables to determine whether there was a significant difference in the harm degrees (the help degrees) among the three stimulation conditions. If a significant main effect was found, *post hoc* analyses (Bonferroni) within ANOVA were run to identify specific differences.

In addition, we also tested for a possible effect of demographic characteristics (sex, age, annual family income, and monthly consumption) on the dependent variables (frame effect scores, harm degrees, and help degrees) when entered in these models as covariates.

**Figure 3:**
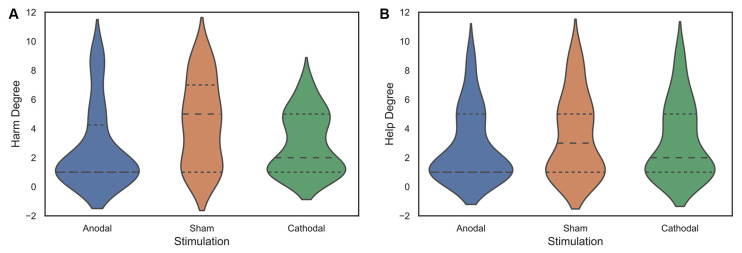
Data of the harm degree **(A)** and help degree **(B)** for all participants under three stimulation conditions.

**Figure 4:**
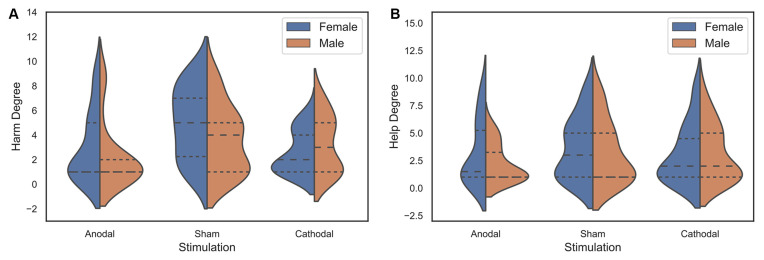
Data of the harm degree **(A)** and help degree **(B)** for females and males under three stimulation conditions.

SPSS software was used to evaluate all data statistically (version 26, SPSS Inc., Chicago, IL, USA). The significance level for all analyses was set at 0.05. Means (M) and standard error (SE) of the data for the social framing effects under three stimulation conditions are shown in Tables s 2, 3. Violin plots of the data for the social framing effects under three stimulation conditions are presented in Figures 3, 4.

**Table 2:** Means (M) and standard error (SE) of the data for the social framing effects under three conditions.

Stimulation	Anodal		Cathodal		Sham	
Frame	M	SE	M	SE	M	SE
Harm Frame	2.646	0.391	2.938	0.294	4.362	0.415
Help Frame	2.750	0.347	3.125	0.368	3.468	0.398
Frame Effect Score	−0.056	0.469	0.012	0.048	0.123	0.041

**Table 3:** Means (M) and standard error (SE) of the data for sex effects of the social framing effects under three conditions.

Stimulation		Anodal		Cathodal		Sham	
Frame		M	SE	M	SE	M	SE
Harm Frame	Male	2.292	0.537	3.240	0.456	3.619	0.587
	Female	3.000	0.571	2.609	0.360	4.962	0.564
Help Frame	Male	2.250	0.347	3.160	0.506	3.238	0.601
	Female	3.250	0.591	3.087	0.548	3.654	0.538
Frame Effect Score	Male	−0.067	0.086	0.033	0.060	0.056	0.068
	Female	−0.0453	0.039	−0.011	0.077	0.177	0.049

## Results

### Behavioural Results

To support the claims on the sham group, we made a comparison between the control group and the sham group. First, we examined whether frame manipulation (within-subjects) was effective in the control group. The Wilcoxon test was used, and the results showed that the harm degree was significantly higher than the help degree for all participants in the control group (*z* = −2.623, *p* = 0.009, *η*^2^ = 0.149) and female participants (*z* = −2.228, *p* = 0.026, *η*^2^ = 0.206) but not for male participants (*z* = −1.414, *p* = 0.157, *η*^2^ = 0.091).

Then, to test whether there was a significant difference between the control group and sham group, an ANOVA with tDCS stimulation type (none and sham), and sex as “between-subjects” factors, and the frame effect score (harm degree, help degree) as dependent variables was used, and the results showed that neither a significant main effect for tDCS stimulation type [frame effect score: *F*_(1,89)_ = 0.778, *p* = 0.380, partial *η*^2^ = 0.009; harm degree: *F*_(1,89)_ = 3.550, *p* = 0.063, partial *η*^2^ = 0.038; help degree: *F*_(1,89)_ = 2.849, *p* = 0.095, partial *η*^2^ = 0.031 ] or sex [frame effect score: *F*_(1,89)_ = 2.311, *p* = 0.132, partial *η*^2^ = 0.025; harm degree: *F*_(1,89)_ = 3.587, *p* = 0.061, partial *η*^2^ = 0.039; help degree: *F*_(1,89)_ = 0.887, *p* = 0.349, partial *η*^2^ = 0.010] nor a significant interaction effect involving the frame and stimulation type [frame effect score: *F*_(1,89)_ = 0.878, *p* = 0.351, partial *η*^2^ = 0.010; harm degree: *F*_(1,89)_ = 0.109, *p* = 0.742, partial *η*^2^ = 0.001; help degree: *F*_(1,89)_ = 0.031, *p* = 0.861, partial *η*^2^ < 0.001] was observed.

Moreover, we also tested for a possible effect of demographic characteristics (age, annual family income, and monthly consumption) on the dependent variables (frame effect scores, harm degrees, and help degrees) when entered in these models as covariates. No significant effect was observed.

We found that female participants in the control group showed a social framing effect, whereas male participants showed no social framing effect. These results were consistent with those in the sham group (see “Effects of tDCS Over the VMPFC on Social Framing” section paragraph (1). Furthermore, there was no significant difference between the control group and sham group in frame effect score, harm degree, or help degree, which could support the claims on the sham group. The sham group was used as the base group to test the stimulation effect.

### Effects of tDCS Over the VMPFC on Social Framing

**Figure 5:**
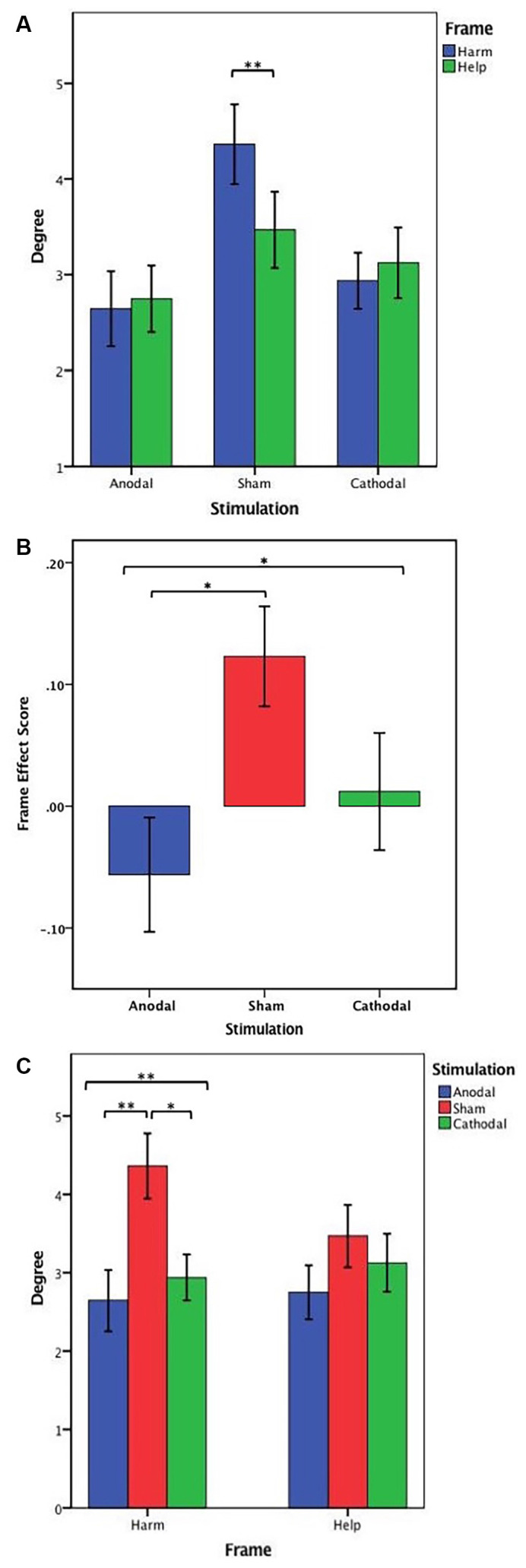
**(A)** Harm degree and help degree under three stimulation conditions. **(B)** Frame effect score in three stimulation conditions. **(C)** Degree in the harm frame and the help frame across three stimulation conditions. Error bar represents standard error. Asterisks indicate a statistically significant difference between the harm degree and the help degree.

First, we examined whether frame manipulation (within-subjects) was effective in the three treatments. The Wilcoxon test was used, and the results showed that the harm degree was significantly higher than the help degree (*z* = −2.603, *p* = 0.009, *η*^2^ = 0.144) in the sham stimulation group but not in the anodal and cathodal stimulation groups (anodal, *z* = −1.178, *p* = 0.239, *η*^2^ = 0.029; cathodal, *z* = −0.196, *p* = 0.845, *η*^2^ = 0.001). The summary presented in Table 2 may be needed to understand these results better. These results meant that the social framing effect was observed in the sham stimulation but not in the anodal and cathodal stimulations (Figure 5A).

Then, to assess whether the social framing effect differed significantly among the three stimulus conditions and to clarify the stimulation effect, the frame effect scores from all stimulation groups were analysed by ANOVA with the tDCS stimulation type (anodal, cathodal, and sham) as a “between-subjects” factor. A significant main effect of stimulation type (*F*_(2,140)_ = 3.941, *p* = 0.022, partial *η*^2^ = 0.053) was observed. *Post hoc* analyses (Bonferroni) revealed that the social framing effect of anodal stimulation (mean = −0.056) was significantly lower than that of sham stimulation (mean = 0.123, *p* = 0.018). Although the social framing effect of cathodal stimulation (mean = 0.026) was lower than that of sham stimulation, the difference was not prominent (*p* = 0.261). Moreover, there was no significant difference in the social framing effect between anodal stimulation and cathodal stimulation (*p* = 0.864; Figure 5B). These results indicated that the activation of the VMPFC decreased the social framing effect.

Furthermore, to explain the difference in the social frame effect under the three stimulation conditions, we conducted an ANOVA with tDCS stimulation type (anodal, cathodal, and sham) as a “between-subjects” factor and the harm degrees as dependent variables. The results showed that the harm degree differed prominently among the three stimulation conditions (*F*_(2,140)_ = 6.122, *p* = 0.003, partial *η*^2^ = 0.080). *Post hoc* analyses (Bonferroni) showed that the harm degree was significantly lower under anodal stimulation (mean = 2.65) than under sham stimulation (mean = 4.36, *p* = 0.004), and the harm degree under cathodal stimulation (mean = 2.94) was also notably lower than that under sham stimulation (*p* = 0.022). Moreover, there was no significant difference in the harm degree between anodal stimulation and cathodal stimulation (*p* = 1.000). Similarly, ANOVA with tDCS stimulation type (anodal, cathodal, and sham) as a “between-subjects” factor and help degree as a dependent variable was conducted to assess whether there was a significant difference in help degree among the three stimulation conditions. The results showed that there was no notable difference in the help degree across the three stimulation conditions (*F*_(2,140)_ = 0.935, *p* = 0.395, partial *η*^2^ = 0.013; Figure 5C). These results indicated a decreased social framing effect due to the increased harm from the deciders but not decreased help.

In addition, we also tested for a possible effect of demographic characteristics (sex, age, annual family income, and monthly consumption) on the dependent variables (frame effect scores, harm degrees, and help degrees) when entered in the models as covariates. No significant effect was observed.

### Sex Effects

We also tested the effect of gender differences on social framing effects under the three tDCS stimulation conditions.

**Figure 6:**
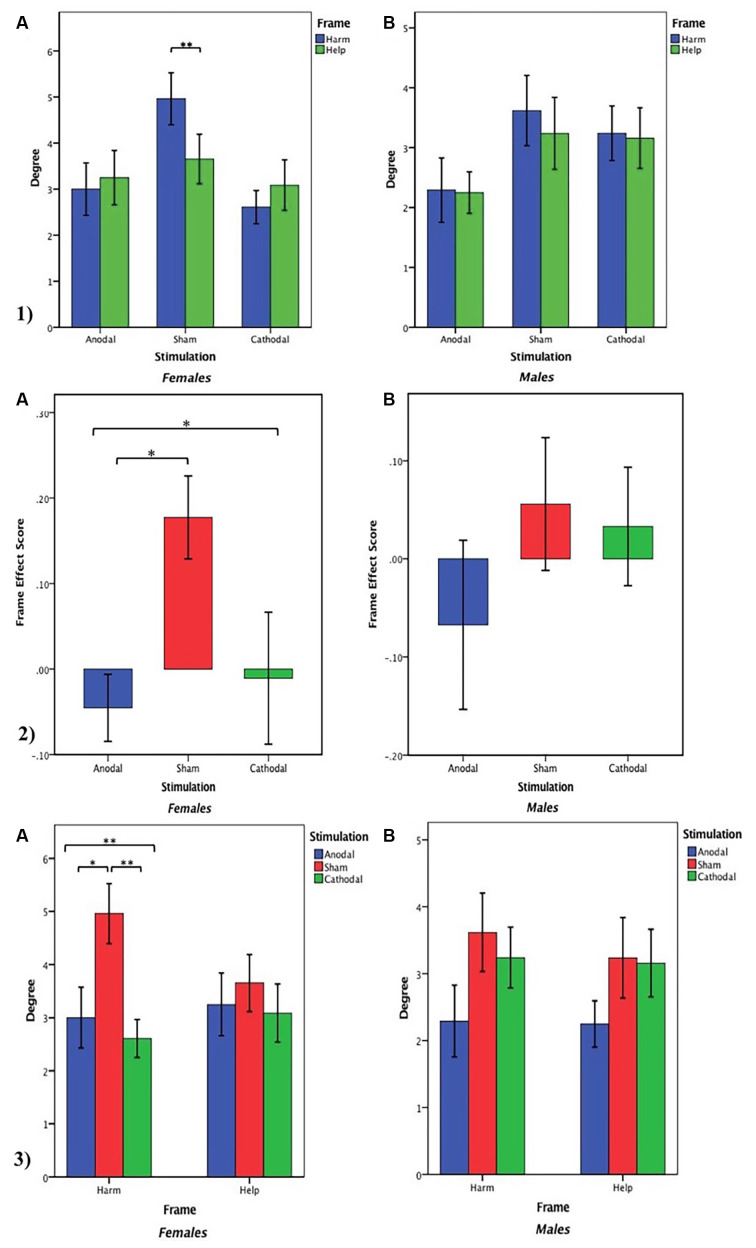
(1) Harm degree and help degree in three stimulation conditions. (2) Frame effect score in three stimulation conditions. (3) Degree in the harm frame and the help frame across three stimulation conditions. **(A)** For females. **(B)** For males. Error bar represents standard error. Asterisks indicate a statistically significant difference between the harm degree and the help degree.

For female participants, first, we examined whether frame manipulation (within-subjects) was effective in the three treatments. The Wilcoxon test was used to analyse the differences between the harm degree and the help degree in different stimulations. We found that the harm degree was significantly higher than the help degree in the sham stimulation group (*z* = −2.877, *p* = 0.004, *η*^2^ = 0.318) but not in the anodal and cathodal stimulation groups (anodal, *z* = −1.540, *p* = 0.124, *η*^2^ = 0.099; cathodal, *z* = −0.704, *p* = 0.481, *η*^2^ = 0.022; Figure 6-1A). Similarly, for male participants, there was no significant difference between the harm degree and the help degree in the three stimulations (anodal, *z* = −0.267, *p* = 0.789, *η*^2^ = 0.003; cathodal, *z* = −0.479, *p* = 0.632, *η*^2^ = 0.009; sham, *z* = −0.848, *p* = 0.396, *η*^2^ = 0.034; Figure 6-1B). The summary presented in Table 3 may be needed to understand these results better. These results implied that the social framing effect for female participants was observed in the sham stimulation but not in the anodal and cathodal stimulations, and there was no social framing effect for male participants under different stimulations.

Then, an ANOVA with the tDCS stimulation type (anodal, cathodal, and sham) as a “between-subjects” factor and the frame effect scores as dependent variables was used to determine whether there was a significant difference in the social framing effects of female participants and male participants among the three stimulation conditions. For female participants, there was a significant main effect of the stimulation type on the social framing effect (*F*_(2,70)_ = 4.690, *p* = 0.012, partial *η*^2^ = 0.118). *Post hoc* analyses (Bonferroni) showed that the social framing effect of anodal stimulation (mean = −0.045) was significantly lower than that of sham stimulation (mean = 0.177, *p* = 0.018). Although the social framing effect of cathodal stimulation (mean = −0.011) was lower than that of sham stimulation, the difference was not prominent (*p* = 0.062). Moreover, there was no significant difference in the social framing effect between the anodal and cathodal stimulations (*p* = 1.000; Figure 6-2A). For male participants, there was no significant main effect of stimulation type on the social framing effect (ANOVA, *F*_(2,67)_ = 0.813, *p* = 0.448, partial *η*^2^ = 0.024; Figure 6-2B). These results indicated that the social framing effect was sensitive to tDCS stimulation over the VMPFC for females but not for male participants.

Furthermore, to explain the difference in the social frame effect under the three stimulation conditions, an ANOVA with tDCS stimulation type (anodal, cathodal, and sham) as a “between-subjects” factor was used to assess whether there was a prominent difference in the harm degree of females among the three stimulation conditions. A significant main effect of stimulation type (*F*_(2,70)_ = 6.142, *p* = 0.003, partial *η*^2^ = 0.149) was observed. *Post hoc* analyses (Bonferroni) showed that the harm degree was prominently lower after receiving anodal stimulation (mean = 3.00) compared with sham stimulation (mean = 4.96, *p* = 0.024). The harm degree of the cathodal stimulation (mean = 2.61) was also notably lower than that of the sham stimulation (*p* = 0.006). There was no significant difference in the harm degree between the anodal and cathodal stimulations (*p* = 1.000). Then, an ANOVA with tDCS stimulation type (anodal, cathodal, and sham) as a “between-subjects” factor was also used to assess whether there was a prominent difference in the help degree of females under the three stimulation conditions. The test results showed that there was no significant main effect of stimulation type on the help degree for females (*F*_(2,70)_ = 0.278, *p* = 0.758, partial *η*^2^ = 0.008; Figure 6-3A). Similarly, there was no significant main effect of stimulation type on the harm degree or the help degree for males (ANOVA, harm degree, *F*_(2,67)_ = 1.679, *p* = 0.194, partial *η*^2^ = 0.048; ANOVA, help degree, *F*_(2,67)_ = 1.277, *p* = 0.285, partial *η*^2^ = 0.037; Figure 6-3B). These results suggested that the harm frame was insensitive to tDCS stimulation on the VMPFC for male participants. Moreover, for the help frame, regardless of gender, the effect of tDCS on the VMPFC was not significant.

In addition, we also tested for a possible effect of demographic characteristics (age, annual family income, and monthly consumption) on the dependent variables (females: frame effect scores, harm degrees and help degrees; males: frame effect scores, harm degrees and help degrees) when entered in these models as covariates. No significant effect was observed.

## Discussion

### VMPFC and the Social Framing Effect

Our results provide evidence that social decision-making is influenced by verbal representation and that the VMPFC plays a role in the social framing effect. In the current study, social framing effects were observed in the control group and the sham stimulation group. Participants’ preference for the costly helping option significantly increased in the harm frame task compared with the help frame task. Anodal stimulation over the VMPFC lowered the social framing effect. Furthermore, both anodal and cathodal stimulation of the VMPFC increased the harm from the participants to the victim but did not reduce the help. Overall, it was confirmed that the VMPFC is important to the social framing effect.

In the current study, the harm frame may induce a stronger sense of moral conflict than the help frame because choosing to harm is a stronger violation of social norms and moral standards than choosing not to help. Therefore, participants did not increase the possibility of harming others in the harm frame; instead, they increased the possibility of not helping others in the help frame. Previous studies (Liu et al., 2020; Fang et al., 2021) showed that the social framing effect was significantly larger than zero in sham treatments, which was consistent with our findings.

Our findings highlighted that the VMPFC plays a role in the social framing effect. Increasing the activity of the VMPFC can reduce the social framework effect because participants preferred harming others to costly helping others. Compared with the help frame, the harm frame also contains intentional harm and is, therefore, more likely to be regarded as “immoral and unacceptable” in moral judgements. Thus, the activation of the VMPFC may reduce the participant’s moral judgement. The decision to be made concerns whether to take somebody’s life with one’s own hands to save multiple people and patients with VMPFC choose the utilitarian option more often than controls (Ciaramelli et al., 2007; Koenigs et al., 2007; Moretto et al., 2010; Thomas et al., 2011). In addition to influencing moral decision-making, there is another possibility that participants are more self-serving when the VMPFC is activated. Participants with stronger VMPFC activity may be dishonest for either self-serving or prosocial benefits (Pornpattananangkul et al., 2018). An increase in selfish motivation for Pareto lies was associated with higher mean-level activity in both the VMPFC and RMPFC (Kim and Kim, 2021). VMPFC patients make more utilitarian decisions because they lack internal markers of emotionally aversive states (Feldmanhall et al., 2013). Furthermore, hurting others may be considered less empathatic. Activation of the VMPFC may reduce the empathy of participants and thus increase harm to the victim or avoid arousing the perception of the pain of others and the appearance of negative results. The VMPFC is an important relay station between cognitive and affective processing (Walter, 2012). The VMPFC may involve an empathy network (Winker et al., 2019). The VMPFC is a key region involving evaluating the similarities and differences distinguishing the mental states of oneself from others (Shamay-Tsoory, 2011). Patients with damage in the VMPFC exhibit impaired empathy because they are unable to generate feelings that guide adaptive decision-making in healthy individuals (Bechara et al., 1996; Anderson et al., 1999; Shamay-Tsoory, 2007). In addition, the path from the amygdala to the VMPFC is hypothesized to be involved in perceiving distress in others and in learning to avoid behaviours that provoke such distress (Rilling and Sanfey, 2011). The disappearance of the social framing effect may also be related to other functions of the VMPFC. The effects of VMPC damage on emotion processing depend on context (Koenigs et al., 2007). Previous studies have shown that VMPFC activation appears to play a crucial role in evaluating the negative consequences of social decision-making (Grossman et al., 2010). In the current study, the cost of preventing one person from receiving a shock or helping one person prevent them from receiving a painful shock is equal to 5 CNY (approximately 0.77 US dollars). Kuss et al. (2015) found that VMPFC activity was observed for non-costly social decisions. Therefore, the possible conclusion is that the relationship between VMPFC activation and social decision-making does not involve a cost decision. A previous study argued that the social framing effect was more substantial under anodal stimulation than under cathodal stimulation over the right TPJ (rTPJ; Liu et al., 2020). However, our results show that activation of the VMPFC reduces the social framing effect. It may be concluded that the social framing effect can be enhanced by tDCS over the rTPJ and can be reduced over the VMPFC. Difficult moral decisions activate the bilateral TPJ and deactivate the VMPFC and OFC (Feldmanhall et al., 2014). However, the inner neural mechanism needs to be further studied.

The data also showed that both anodal and cathodal stimulation of the VMPFC increased harm from the participants to the victim but did not reduce help. It is not clear why both anodal and cathodal stimulations induce participants to change their behaviour in the same direction. However, some studies have shown that this is not an unexpected result. Both anodal and cathodal transcranial direct current stimulation (tDCS) improves semantic processing (Brückner and Kammer, 2017). For bilateral stimuli, the effects of reducing spatial attention were driven by polarity nonspecific compared with sham tDCS (Filmer et al., 2015). It has been reported that 2 mA anodal and cathodal tDCS over the VMPFC promoted self-esteem ratings (Salehinejad et al., 2020). However, the reasons for explaining this phenomenon need to be further studied.

### VMPFC and Sex

One interesting finding is that the social framing effect for female participants was observed but not for male participants in the control group and the sham stimulations. Only female participants induced the social framing effect. Previous literature has suggested that female participants are susceptible to social framing and that male participants are not (Ellingsen et al., 2013), consistent with our finding. The cortical excitability of the VMPFC has a significant effect on females regarding the social framing effect, but the effect was not obvious for males. The harm frame for females was more sensitive to tDCS stimulation over the VMPFC than that for males. Overall, it was confirmed that there are gender differences in the social framing effect. Additionally, there are gender differences in the impact of the VMPFC on the social framing effect.

In the current study, the gender difference in the social framing effect is due to the difference in decision-making between the sexes in the harm frame. Modulating the activity of the VMPFC results in gender differences in the social framing effect, which also stems from changes in females’ decision-making in the harm frame. Therefore, the gender decision-making difference in the harm frame is the key point to produce and change the social framework effect. The gender difference in moral decision-making may explain this difference. Men tend to embrace consequentialist judgement significantly more than women, but only in the case of personal moral dilemmas (Fumagalli et al., 2010). Men showed a stronger preference for utilitarian over deontological judgements than women. Women exhibited stronger deontological inclinations than men (Friesdorf et al., 2015). Women scored higher than men on deontological tendencies, and this difference was enhanced when the deontological choice required refraining from harmful action rather than acting to prevent harm (Armstrong et al., 2019). Gender differences in empathy may be another possible explanation. Female patients showed higher levels of empathy than male patients, both before and after cognitive-behavioural therapy (Lim et al., 2018). Examinations of the neurobiological underpinnings of empathy reveal important quantitative gender differences in the basic networks involved in affective and cognitive forms of empathy, as well as a qualitative divergence between the sexes in how emotional information is integrated to support decision-making processes (Christov-Moore et al., 2014). The above explanation implies that females may be more empathetic or have stronger deontological inclinations than males.

### Limitations

The limitation of the current research is that all the tasks were conducted to examine the participants’ decisions at a cost of 5 CNY and did not examine the decisions made at different costs; therefore, we cannot be sure whether more money would change the decisions of the participants. Another drawback is that we did not conduct experiments on the nonsocial framing effect, which could be used as a control experiment. Hence, future studies may explore the causal relationship between the VMPFC and the nonsocial framing effect. For tDCS, because it can accomplish long-lasting effects and its application is simple, it has been increasingly used. However, one drawback of tDCS is its low focality. The focality could be accomplished by reducing the stimulation electrode size or increasing the reference electrode size (Nitsche et al., 2007). Therefore, a 3 cm*3 cm stimulation electrode and a 5 cm*7 cm reference electrode were used in the current study to enhance the focality (Frings et al., 2018) and reduce the area of possible influence over oFC and dlPFC. What we know at present is that the social framework experiment method used in this article has only been experimented with in China, so we do not have information about the results of this experiment in other countries or regions. However, the topic was interesting, and further study is necessary. Prior to further studies, we might guess that the difference in cultural background may not affect the results of the experiment.

## Conclusions

Our research confirmed the social framing effect once again. We found that modulating the activity of the VMPFC with tDCS lowered the social framing effect. Furthermore, participants tended to increase harm to others but did not reduce help to others after receiving anodal tDCS over the VMPFC, explaining the variation in the social framing effect between the treatments. Moreover, we found that there are gender differences in the social framing effect. The harm frame for females was more sensitive to tDCS stimulation on the VMPFC than that for males. In general, our findings revealed that modulating the activity of the VMPFC with tDCS alters the social framing effect and provide new evidence for the neural basis of social framing effects.

## Data Availability Statement

The original contributions presented in the study are included in the article/Supplementary Material, further inquiries can be directed to the corresponding author.

## Ethics Statement

The studies involving human participants were reviewed and approved by Zhejiang University of Finance and Economics ethics committee. Written informed consent to participate in this study was provided by the participants’ legal guardian/next of kin.

## Author Contributions

YC, PY, XL, WG and HY designed experiments. YC, PY, LZ, and WG performed the experiments. YC and WG analysed the data and wrote the manuscript. YC and QS drew figures. All authors contributed to the article and approved the submitted version.

## Conflict of Interest

The authors declare that the research was conducted in the absence of any commercial or financial relationships that could be construed as a potential conflict of interest.

## Publisher’s Note

All claims expressed in this article are solely those of the authors and do not necessarily represent those of their affiliated organizations, or those of the publisher, the editors and the reviewers. Any product that may be evaluated in this article, or claim that may be made by its manufacturer, is not guaranteed or endorsed by the publisher.

## References

[B1] Adolphs R. (2010). Conceptual challenges and directions for social neuroscience. Neuron 65, 752–767. 10.1016/j.neuron.2010.03.006 20346753 PMC2887730

[B2] Anderson S. W. Bechara A. Damasio H. Tranel D. Damasio A. R. (1999). Impairment of social and moral behavior related to early damage in human prefrontal cortex. Nat. Neurosci. 2, 1032–1037. 10.1038/14833 10526345

[B3] Armstrong J. Friesdorf R. Conway P. (2019). Clarifying gender differences in moral dilemma judgments: the complementary roles of harm aversion and action aversion. Soc. Psychol. Personal. Sci. 10, 353–363. 10.1177/1948550618755873

[B4] Baliki M. N. Petre B. Torbey S. Herrmann K. M. Huang L. Schnitzer T. J.. (2012). Corticostriatal functional connectivity predicts transition to chronic back pain. Nat. Neurosci. 15, 1117–1119. 10.1038/nn.3153 22751038 PMC3411898

[B5] Bechara A. Tranel D. Damasio H. Damasio A. R. (1996). Failure to respond autonomically to anticipated future outcomes following damage to prefrontal cortex. Cereb. Cortex 6, 215–225. 10.1093/cercor/6.2.215 8670652

[B6] Benford R. D. Snow D. A. (2000). Framing processes and social movements: an overview and assessment. Ann. Rev. Soc. 26, 611–639. 10.1146/annurev.soc.26.1.611

[B7] Brückner S. Kammer T. (2017). Both anodal and cathodal transcranial direct current stimulation improves semantic processing. Neuroscience 343, 269–275. 10.1016/j.neuroscience.2016.12.015 28003159

[B8] Chen D. L. Schonger M. Wickens C. (2016). oTree—an open-source platform for laboratory, online and field experiments. J. Behav. Exp. Finance 9, 88–97. 10.1016/j.jbef.2015.12.001

[B9] Christov-Moore L. Simpson E. A. Coude G. Grigaityte K. Iacoboni M. Ferrari P. F.. (2014). Empathy: gender effects in brain and behavior. Neurosci. Biobehav. Rev. 46, 604–627. 10.1016/j.neubiorev.2014.09.001 25236781 PMC5110041

[B10] Ciaramelli E. Muccioli M. Ladavas E. Di Pellegrino G. (2007). Selective deficit in personal moral judgment following damage to ventromedial prefrontal cortex. Soc. Cogn. Affect Neurosci. 2, 84–92. 10.1093/scan/nsm001 18985127 PMC2555449

[B11] De Martino B. Kumaran D. Seymour B. Dolan R. J. (2006). Frames, biases and rational decision-making in the human brain. Science 313, 684–687. 10.1126/science.1128356 16888142 PMC2631940

[B12] Deppe M. Schwindt W. Kramer J. Kugel H. Plassmann H. Kenning P.. (2005). Evidence for a neural correlate of a framing effect: bias-specific activity in the ventromedial prefrontal cortex during credibility judgments. Brain Res. Bull. 67, 413–421. 10.1016/j.brainresbull.2005.06.017 16216688

[B13] Dymond A. M. Coger R. W. Serafetinides E. A. (1975). Intracerebral current levels in man during electrosleep therapy. Biol. Psychiatry 10, 101–104. 1120172

[B14] Eisenberger N. I. Master S. L. Inagaki T. K. Taylor S. E. Shirinyan D. Lieberman M. D.. (2011). Attachment figures activate a safety signal-related neural region and reduce pain experience. Proc. Natl. Acad. Sci. U S A 108, 11721–11726. 10.1073/pnas.1108239108 21709271 PMC3136329

[B15] Ellingsen T. Johannesson M. Mollerstrom J. Munkhammar S. (2012). Social framing effects: preferences or beliefs? Games Econom. Behav. 76, 117–130. 10.1016/j.geb.2012.05.007

[B16] Ellingsen T. Johannesson M. Mollerstrom J. Munkhammar S. (2013). Gender differences in social framing effects. Econom. Lett. 118, 470–472. 10.1016/j.econlet.2012.12.010

[B17] Fang C. Jiamiao Y. Ruolei G. Jie L. (2021). Functional connectivities of the right temporoparietal junction and moral network predict social framing effect: evidence from resting-state fMRI. Acta Psychol. Sin. 53:55. 10.3724/sp.j.1041.2021.00055

[B18] Faul F. Erdfelder E. Buchner A. Lang A.-G. (2009). Statistical power analyses using G* Power 3.1: Tests for correlation and regression analyses. Behav. Res. Methods 41, 1149–1160. 10.3758/BRM.41.4.1149 19897823

[B19] Feldmanhall O. Dalgleish T. Mobbs D. (2013). Alexithymia decreases altruism in real social decisions. Cortex 49, 899–904. 10.1016/j.cortex.2012.10.015 23245426

[B20] Feldmanhall O. Mobbs D. Dalgleish T. (2014). Deconstructing the brain’s moral network: dissociable functionality between the temporoparietal junction and ventro-medial prefrontal cortex. Soc. Cogn. Affect. Neurosci. 9, 297–306. 10.1093/scan/nss139 23322890 PMC3980797

[B21] Filmer H. L. Dux P. E. Mattingley J. B. (2015). Dissociable effects of anodal and cathodal tDCS reveal distinct functional roles for right parietal cortex in the detection of single and competing stimuli. Neuropsychologia 74, 120–126. 10.1016/j.neuropsychologia.2015.01.038 25637773

[B22] Fossati P. (2012). Neural correlates of emotion processing: from emotional to social brain. Eur. Neuropsychopharmacol. 22, S487–S491. 10.1016/j.euroneuro.2012.07.008 22959113

[B23] Friesdorf R. Conway P. Gawronski B. (2015). Gender differences in responses to moral dilemmas: a process dissociation analysis. Pers. Soc. Psychol. Bull. 41, 696–713. 10.1177/0146167215575731 25840987

[B24] Frings C. Brinkmann T. Friehs M. A. Van Lipzig T. (2018). Single session tDCS over the left DLPFC disrupts interference processing. Brain Cogn. 120, 1–7. 10.1016/j.bandc.2017.11.005 29202318

[B25] Fumagalli M. Ferrucci R. Mameli F. Marceglia S. Mrakic-Sposta S. Zago S.. (2010). Gender-related differences in moral judgments. Cogn. Process. 11, 219–226. 10.1007/s10339-009-0335-2 19727878

[B26] Gandiga P. C. Hummel F. C. Cohen L. G. (2006). Transcranial DC stimulation (tDCS): a tool for double-blind sham-controlled clinical studies in brain stimulation. Clin. Neurophysiol. 117, 845–850. 10.1016/j.clinph.2005.12.003 16427357

[B27] Gonzalez C. Dana J. Koshino H. Just M. (2005). The framing effect and risky decisions: examining cognitive functions with fMRI. J. Econom. Psychol. 26, 1–20. 10.1016/j.joep.2004.08.004

[B28] Grossman M. Eslinger P. J. Troiani V. Anderson C. Avants B. Gee J. C.. (2010). The role of ventral medial prefrontal cortex in social decisions: converging evidence from fMRI and frontotemporal lobar degeneration. Neuropsychologia 48, 3505–3512. 10.1016/j.neuropsychologia.2010.07.036 20691197 PMC2949451

[B29] Hare T. A. Camerer C. F. Rangel A. (2009). Self-control in decision-making involves modulation of the vmPFC valuation system. Science 324, 646–648. 10.1126/science.1168450 19407204

[B30] Hartley C. A. Phelps E. A. (2010). Changing fear: the neurocircuitry of emotion regulation. Neuropsychopharmacology 35, 136–146. 10.1038/npp.2009.121 19710632 PMC3055445

[B31] Hashmi J. A. Baliki M. N. Huang L. Baria A. T. Torbey S. Hermann K. M.. (2013). Shape shifting pain: chronification of back pain shifts brain representation from nociceptive to emotional circuits. Brain 136, 2751–2768. 10.1093/brain/awt211 23983029 PMC3754458

[B32] Hernandez M. Denburg N. L. Tranel D. (2009). “A neuropsychological perspective on the role of the prefrontal cortex in reward processing and decision-making,” in Handbook of Reward and Decision Making, (Amsterdam, Netherlands: Elsevier), 291–306.

[B33] Hutcherson C. A. Montaser-Kouhsari L. Woodward J. Rangel A. (2015). Emotional and utilitarian appraisals of moral dilemmas are encoded in separate areas and integrated in ventromedial prefrontal cortex. J. Neurosci. 35, 12593–12605. 10.1523/JNEUROSCI.3402-14.2015 26354924 PMC4563040

[B34] Hynes C. A. Baird A. A. Grafton S. T. (2006). Differential role of the orbital frontal lobe in emotional versus cognitive perspective-taking. Neuropsychologia 44, 374–383. 10.1016/j.neuropsychologia.2005.06.011 16112148

[B35] Kim J. Kim H. (2021). Neural representation in MPFC reveals hidden selfish motivation in white lies. J. Neurosci. 41, 5937–5946. 10.1523/JNEUROSCI.0088-21.2021 34059555 PMC8265801

[B36] Kim M. J. Gee D. G. Loucks R. A. Davis F. C. Whalen P. J. (2011). Anxiety dissociates dorsal and ventral medial prefrontal cortex functional connectivity with the amygdala at rest. Cereb. Cortex 21, 1667–1673. 10.1093/cercor/bhq237 21127016 PMC3116741

[B37] Koenigs M. Young L. Adolphs R. Tranel D. Cushman F. Hauser M.. (2007). Damage to the prefrontal cortex increases utilitarian moral judgements. Nature 446, 908–911. 10.1038/nature05631 17377536 PMC2244801

[B38] Krajbich I. Adolphs R. Tranel D. Denburg N. L. Camerer C. F. (2009). Economic games quantify diminished sense of guilt in patients with damage to the prefrontal cortex. J. Neurosci. 29, 2188–2192. 10.1523/JNEUROSCI.5086-08.2009 19228971 PMC2646169

[B39] Kuo M. F. Paulus W. Nitsche M. A. (2014). Therapeutic effects of non-invasive brain stimulation with direct currents (tDCS) in neuropsychiatric diseases. NeuroImage 85, 948–960. 10.1016/j.neuroimage.2013.05.117 23747962

[B40] Kuss K. Falk A. Trautner P. Montag C. Weber B. Fliessbach K.. (2015). Neuronal correlates of social decision making are influenced by social value orientation-an fMRI study. Front. Behav. Neurosci. 9:40. 10.3389/fnbeh.2015.00040 25759643 PMC4338788

[B41] Lefaucheur J.-P. Antal A. Ayache S. S. Benninger D. H. Brunelin J. Cogiamanian F.. (2017). Evidence-based guidelines on the therapeutic use of transcranial direct current stimulation (tDCS). Clin. Neurophysiol. 128, 56–92. 10.1016/j.clinph.2016.10.087 27866120

[B42] Li Y. Wang J. Ye H. Luo J. (2020). Modulating the activity of vmpfc regulates informational social conformity: a tDCS study. Front. Psychol. 11:566977. 10.3389/fpsyg.2020.566977 33041931 PMC7527649

[B43] Lim J. A. Choi S. H. Lee W. J. Jang J. H. Moon J. Y. Kim Y. C.. (2018). Cognitive-behavioral therapy for patients with chronic pain: Implications of gender differences in empathy. Medicine (Baltimore) 97:e10867. 10.1097/MD.0000000000010867 29879022 PMC5999451

[B44] Liu J. Gu R. Liao C. Lu J. Fang Y. Xu P.. (2020). The neural mechanism of the social framing effect: evidence from fMRI and tDCS studies. J. Neurosci. 40, 3646–3656. 10.1523/JNEUROSCI.1385-19.2020 32238480 PMC7189763

[B45] Mahoney K. T. Buboltz W. Levin I. P. Doverspike D. Svyantek D. J. (2011). Individual differences in a within-subjects risky-choice framing study. Personal. Individual Diff. 51, 248–257. 10.1016/j.paid.2010.03.035

[B46] Meyer-Lindenberg A. Tost H. (2012). Neural mechanisms of social risk for psychiatric disorders. Nat. Neurosci. 15, 663–668. 10.1038/nn.3083 22504349

[B47] Milad M. R. Quirk G. J. (2002). Neurons in medial prefrontal cortex signal memory for fear extinction. Nature 420, 70–74. 10.1038/nature01138 12422216

[B48] Mobbs D. Yu R. Meyer M. Passamonti L. Seymour B. Calder A. J.. (2009). A key role for similarity in vicarious reward. Science 324:900. 10.1126/science.1170539 19443777 PMC2839480

[B49] Morelli S. A. Rameson L. T. Lieberman M. D. (2014). The neural components of empathy: predicting daily prosocial behavior. Soc. Cogn. Affect. Neurosci. 9, 39–47. 10.1093/scan/nss088 22887480 PMC3871722

[B50] Moretto G. Ladavas E. Mattioli F. Di Pellegrino G. (2010). A psychophysiological investigation of moral judgment after ventromedial prefrontal damage. J. Cogn. Neurosci. 22, 1888–1899. 10.1162/jocn.2009.21367 19925181

[B51] Moretto G. Sellitto M. Di Pellegrino G. (2013). Investment and repayment in a trust game after ventromedial prefrontal damage. Front. Hum. Neurosci. 7:593. 10.3389/fnhum.2013.00593 24093013 PMC3782646

[B52] Nejati V. Majdi R. Salehinejad M. A. Nitsche M. A. (2021). The role of dorsolateral and ventromedial prefrontal cortex in the processing of emotional dimensions. Sci. Rep. 11:1971. 10.1038/s41598-021-81454-7 33479323 PMC7819980

[B53] Nitsche M. A. Paulus W. (2000). Excitability changes induced in the human motor cortex by weak transcranial direct current stimulation. J. Physiol. 527, 633–639. 10.1111/j.1469-7793.2000.t01-1-00633.x 10990547 PMC2270099

[B54] Nitsche M. A. Doemkes S. Karakose T. Antal A. Liebetanz D. Lang N.. (2007). Shaping the effects of transcranial direct current stimulation of the human motor cortex. J. Neurophysiol. 97, 3109–3117. 10.1152/jn.01312.2006 17251360

[B55] Nitsche M. A. Liebetanz D. Lang N. Antal A. Tergau F. Paulus W.. (2003). Safety criteria for transcranial direct current stimulation (tDCS) in humans. Clin. Neurophysiol. 114, 2220–2222. 10.1016/s1388-2457(03)00235-9 14580622

[B56] Pan Z. Kosicki G. M. (1993). Framing analysis: An approach to news discourse. Political Commun. 10, 55–75.

[B57] Peng J. Li H. Miao D. Feng X. Xiao W. (2013). Five different types of framing effects in medical situation: a preliminary exploration. Iran. Red Crescent Med. J. 15, 161–165. 10.5812/ircmj.8469 23682330 PMC3652505

[B58] Phelps E. A. Delgado M. R. Nearing K. I. Ledoux J. E. (2004). Extinction learning in humans: role of the amygdala and vmPFC. Neuron 43, 897–905. 10.1016/j.neuron.2004.08.042 15363399

[B59] Pornpattananangkul N. Zhen S. Yu R. (2018). Common and distinct neural correlates of self-serving and prosocial dishonesty. Hum. Brain Mapp. 39, 3086–3103. 10.1002/hbm.24062 29582512 PMC6866300

[B60] Rilling J. K. Sanfey A. G. (2011). The neuroscience of social decision-making. Annu. Rev. Psychol. 62, 23–48. 10.1146/annurev.psych.121208.131647 20822437

[B61] Roy M. Shohamy D. Wager T. D. (2012). Ventromedial prefrontal-subcortical systems and the generation of affective meaning. Trends Cogn. Sci. 16, 147–156. 10.1016/j.tics.2012.01.005 22310704 PMC3318966

[B62] Salehinejad M. A. Nejati V. Nitsche M. A. (2020). Neurocognitive correlates of self-esteem: from self-related attentional bias to involvement of the ventromedial prefrontal cortex. Neurosci. Res. 161, 33–43. 10.1016/j.neures.2019.12.008 31837992

[B63] Sangha S. Robinson P. D. Greba Q. Davies D. A. Howland J. G. (2014). Alterations in reward, fear and safety cue discrimination after inactivation of the rat prelimbic and infralimbic cortices. Neuropsychopharmacology 39, 2405–2413. 10.1038/npp.2014.89 24727732 PMC4138751

[B64] Saxe R. (2006). Uniquely human social cognition. Curr. Opin. Neurobiol. 16, 235–239. 10.1016/j.conb.2006.03.001 16546372

[B65] Scheufele D. A. (1999). Framing as a theory of media effects. J. Commun. 49, 103–122.

[B66] Schiller D. Levy I. Niv Y. Ledoux J. E. Phelps E. A. (2008). From fear to safety and back: reversal of fear in the human brain. J. Neurosci. 28, 11517–11525. 10.1523/JNEUROSCI.2265-08.2008 18987188 PMC3844784

[B67] Shamay-Tsoory S. G. (2007). “Impaired empathy following ventromedial prefrontal brain damage,” in Empathy in Mental Illness, (Cambridge, United Kingdom: Cambridge University Press), 89–110.

[B68] Shamay-Tsoory S. G. (2011). The neural bases for empathy. Neuroscientist 17, 18–24. 10.1177/1073858410379268 21071616

[B69] Shön D. Rein M. (1994). Frame Reflection: Toward the Resolution of Intractable Policy Controversies. New York, NY: Basic Books.

[B70] Silveira S. Fehse K. Vedder A. Elvers K. Hennig-Fast K. (2015). Is it the picture or is it the frame? an fMRI study on the neurobiology of framing effects. Front. Hum. Neurosci. 9:528. 10.3389/fnhum.2015.00528 26528161 PMC4602085

[B71] Sip K. E. Smith D. V. Porcelli A. J. Kar K. Delgado M. R. (2015). Social closeness and feedback modulate susceptibility to the framing effect. Soc. Neurosci. 10, 35–45. 10.1080/17470919.2014.944316 25074501 PMC4250444

[B72] Sotres-Bayon F. Quirk G. J. (2010). Prefrontal control of fear: more than just extinction. Curr. Opin. Neurobiol. 20, 231–235. 10.1016/j.conb.2010.02.005 20303254 PMC2878722

[B73] Talmi D. Hurlemann R. Patin A. Dolan R. J. (2010). Framing effect following bilateral amygdala lesion. Neuropsychologia 48, 1823–1827. 10.1016/j.neuropsychologia.2010.03.005 20227427 PMC2877879

[B74] Tannen D. (1993). Framing in Discourse. Oxford, United Kingdom: Oxford University Press on Demand.

[B75] Thomas B. C. Croft K. E. Tranel D. (2011). Harming kin to save strangers: further evidence for abnormally utilitarian moral judgments after ventromedial prefrontal damage. J. Cogn. Neurosci. 23, 2186–2196. 10.1162/jocn.2010.21591 20946057 PMC3234136

[B76] Triandafyllidou A. Fotiou A. (1998). Sustainability and modernity in the european union: a frame theory approach to policy-making. Soc. Res. Online 3, 60–75.

[B77] Tversky A. Kahneman D. (1981). The framing of decisions and the psychology of choice. Science 211, 453–458. 10.1126/science.7455683 7455683

[B78] Van Kerkhof L. W. Damsteegt R. Trezza V. Voorn P. Vanderschuren L. J. (2013). Social play behavior in adolescent rats is mediated by functional activity in medial prefrontal cortex and striatum. Neuropsychopharmacology 38, 1899–1909. 10.1038/npp.2013.83 23568326 PMC3746695

[B79] Van Kerkhof L. W. Trezza V. Mulder T. Gao P. Voorn P. Vanderschuren L. J. (2014). Cellular activation in limbic brain systems during social play behaviour in rats. Brain Struct. Funct. 219, 1181–1211. 10.1007/s00429-013-0558-y 23670540 PMC3770763

[B80] Vollm B. A. Taylor A. N. Richardson P. Corcoran R. Stirling J. Mckie S.. (2006). Neuronal correlates of theory of mind and empathy: a functional magnetic resonance imaging study in a nonverbal task. NeuroImage 29, 90–98. 10.1016/j.neuroimage.2005.07.022 16122944

[B81] Walter H. (2012). Social cognitive neuroscience of empathy: concepts, circuits and genes. Emotion Rev. 4, 9–17. 10.1177/1754073911421379

[B82] Winker C. Rehbein M. A. Sabatinelli D. Dohn M. Maitzen J. Roesmann K.. (2019). Noninvasive stimulation of the ventromedial prefrontal cortex indicates valence ambiguity in sad compared to happy and fearful face processing. Front. Behav. Neurosci. 13:83. 10.3389/fnbeh.2019.00083 31156403 PMC6532016

[B83] Zheng H. Huang D. Chen S. Wang S. Guo W. Luo J.. (2016). Modulating the activity of ventromedial prefrontal cortex by anodal tDCS enhances the trustee’s repayment through altruism. Front. Psychol. 7:1437. 10.3389/fpsyg.2016.01437 27713721 PMC5031609

[B84] Zheng H. Wang X. T. Zhu L. (2010). Framing effects: behavioral dynamics and neural basis. Neuropsychologia 48, 3198–3204. 10.1016/j.neuropsychologia.2010.06.031 20600178

